# Supplementary material for: Multi‐dataset identification of innovative feature genes and molecular mechanisms in keratoconus
Source: J Cell Mol Med. 2024 Sep 19;28(18):e70079. doi: 10.1111/jcmm.70079 (PMC11412914; doi:10.1111/jcmm.70079)
Supplement: Supplementary file 2 — Material S2. [file JCMM-28-e70079-s002.zip › 7.GSEA analysis/1.ARL11/KEGG_HISTIDINE_METABOLISM.html]

Details for gene set KEGG\_HISTIDINE\_METABOLISM[GSEA]

|  || Dataset | ARL11.group.cls#h\_versus\_l |
| Phenotype | group.cls#h\_versus\_l |
| Upregulated in class | h |
| GeneSet | KEGG\_HISTIDINE\_METABOLISM |
| Enrichment Score (ES) | 0.34375665 |
| Normalized Enrichment Score (NES) | 0.92133135 |
| Nominal p-value | 0.5632184 |
| FDR q-value | 1.0 |
| FWER p-Value | 1.0 |
Table: GSEA Results Summary

  

Fig 1: Enrichment plot: KEGG\_HISTIDINE\_METABOLISM      
 Profile of the Running ES Score & Positions of GeneSet Members on the Rank Ordered List

  

| SYMBOL | TITLE | RANK IN GENE LIST | RANK METRIC SCORE | RUNNING ES | CORE ENRICHMENT || 1 | HNMT | na | 64 | 0.713 | 0.1549 | Yes |
| 2 | HDC | na | 152 | 0.607 | 0.2859 | Yes |
| 3 | MAOB | na | 1064 | 0.382 | 0.3438 | Yes |
| 4 | CNDP1 | na | 4027 | 0.200 | 0.3024 | No |
| 5 | TRMT11 | na | 6943 | 0.113 | 0.2433 | No |
| 6 | FTCD | na | 10264 | 0.057 | 0.1601 | No |
| 7 | METTL2B | na | 10273 | 0.057 | 0.1723 | No |
| 8 | LCMT2 | na | 10722 | 0.051 | 0.1706 | No |
| 9 | AOC1 | na | 10836 | 0.049 | 0.1782 | No |
| 10 | ALDH7A1 | na | 10913 | 0.048 | 0.1867 | No |
| 11 | ALDH3B1 | na | 11840 | 0.038 | 0.1683 | No |
| 12 | MAOA | na | 12281 | 0.034 | 0.1630 | No |
| 13 | ALDH3A2 | na | 13283 | 0.025 | 0.1396 | No |
| 14 | ALDH3B2 | na | 13954 | 0.019 | 0.1245 | No |
| 15 | LCMT1 | na | 19341 | -0.019 | -0.0265 | No |
| 16 | ALDH3A1 | na | 20262 | -0.026 | -0.0473 | No |
| 17 | DDC | na | 23583 | -0.057 | -0.1305 | No |
| 18 | ALDH1B1 | na | 25126 | -0.078 | -0.1578 | No |
| 19 | ALDH9A1 | na | 25157 | -0.078 | -0.1415 | No |
| 20 | AMDHD1 | na | 25699 | -0.086 | -0.1382 | No |
| 21 | METTL6 | na | 26386 | -0.096 | -0.1368 | No |
| 22 | HEMK1 | na | 28577 | -0.145 | -0.1679 | No |
| 23 | ALDH1A3 | na | 28596 | -0.146 | -0.1362 | No |
| 24 | ALDH2 | na | 31150 | -0.226 | -0.1601 | No |
| 25 | UROC1 | na | 31193 | -0.228 | -0.1112 | No |
| 26 | HAL | na | 31694 | -0.248 | -0.0711 | No |
| 27 | ACY3 | na | 32177 | -0.268 | -0.0260 | No |
| 28 | ASPA | na | 34196 | -0.452 | 0.0153 | No |
Table: GSEA details [plain text format]

  

Fig 2: KEGG\_HISTIDINE\_METABOLISM      
 Blue-Pink O' Gram in the Space of the Analyzed GeneSet

  

Fig 3: KEGG\_HISTIDINE\_METABOLISM: Random ES distribution      
 Gene set null distribution of ES for **KEGG\_HISTIDINE\_METABOLISM**
